# Organ preservation, for rectal cancer: general overview of the latest data from phase III randomized trials

**DOI:** 10.2340/1651-226X.2025.41057

**Published:** 2025-01-27

**Authors:** Syrine Ben Dhia, Damien Chauvière, Diana Mitrea, Renaud Schiappa, Tanguy Pace Loscos, Emmanuel Chamorey, David Baron

**Affiliations:** a Department of Radiotherapy, Antoine Lacassagne Center, Nice, France; b Department of Clinical Research and Innovation, Antoine Lacassagne Center, Nice, France; c Department of Epidemiology, Biostatistics and Health Data, Centre Antoine Lacassagne, University of Côte d’Azur, Nice, France

**Keywords:** Rectal cancer, organ preservation, contact x-ray therapy

## Abstract

**Introduction:**

Organ preservation (OP) strategies are gaining interest in improving the quality of life in the management of rectal cancer, particularly for tumors located in the distal or middle rectum. The optimal OP protocol is still not standardized and relies on randomized trials. This review summarizes past and ongoing studies on OP protocols for adenocarcinoma of the distal and middle rectum.

**Method:**

We searched for articles and abstracts on randomized clinical trials investigating OP approaches for rectal cancer, including data presented at the LUCARRE Congress held in Nice on November 25, 2023, covering ongoing and recently published trials on rectal preservation.

**Results:**

Our review’s findings are presented in four tables: the first evaluates key trials with overall survival (OS) as the primary endpoint; the second provides an overview of past Phase III trials; the third reviews Phase II/III trials that specifically focus on local excisions (LE); and finally, the fourth summarizes ongoing trials. Each table is accompanied by detailed comments elucidating the significance and implications of the presented data, alongside a review of current guidelines.

**Interpretation:**

We highlight the growing interest in OP strategies for rectal cancer management to enhance patients’ quality of life. Despite the lack of international consensus on the optimal OP protocol, past and ongoing randomized trials provide valuable findings into the evolving management strategies of rectal cancer treatment. The presented data supports the role of randomized phase III trials to provide evidence for a change in clinical practice.

## Introduction

Rectal cancer is a prevalent worldwide health concern that causes significant mortality and morbidity with the primary treatment modality being radical surgery, advocated by W. Heald as Total Mesorectal Excision (TME) [1]. The classification of non-metastatic rectal adenocarcinomas into early or locally advanced stages remains controversial in the literature.

This classification is based on clinical examination, endoscopic rectal ultrasound findings (ERUS), and magnetic resonance imaging (MRI). According to the latest European Society for Medical Oncology (ESMO) guidelines and the 8^th^ edition of the TNM classification [2, 3] ‘very early’ rectal cancers are defined as cT1 sm1 N0 (on ERUS and MRI), while ‘early rectal cancers’ are characterized by tumors classified as cT1–cT2; cT3a/b if located in the mid or upper rectum, N0 (or cN1 if in the upper rectum), with a clear mesorectal fascia (MRF) and no evidence of extramural vascular invasion (EMVI). The term ‘locally advanced rectal cancer’ (LARC) typically refers to tumors with a higher risk of local recurrence, generally T3 and T4 lesions, which may benefit from neoadjuvant therapy. Tumors with a threatened circumferential resection margin (CRM), including some T2 lesions, also fall under this category [4]. However, criteria vary across studies. Based on the methodology of the OPERA trial [5], we considered tumor size, which led to the establishment of two subgroups according to a cutoff of 3 cm; the distinction between early and locally advanced disease is assessed based on tumor diameter, volume, and circumferential extent.

The risk of lymph node involvement increases with tumor size as demonstrated in the TAU-TEM trial, which reported a 20% rate of lymph node involvement in tumors smaller than 4 cm [6]. This finding supports the potential consideration of neoadjuvant therapy even for patients with early-stage rectal cancers.

When combining neoadjuvant radiotherapy and chemotherapy with TME, local control is achieved in close to 95% of cases. Five-year overall survival (OS) in recent randomized trials for LARC is between 85 and 90% [7–10]. Anterior resection is the most frequent radical surgery performed but the quality of the bowel movement is often poor as measured by the Low Anterior Resection Syndrome (LARS) score [11] despite anal sphincter preservation. To improve the quality of life of these patients, especially with tumors located in the distal or middle rectum, there is an increasing interest in conservative rectal strategies and organ preservation (OP). The optimal protocol for OP is not yet known. Randomized trials with OP as the main endpoint can give high-level evidence for the relevance of any past or new regimen [12].

In this review, we present an overview of past published trials and ongoing studies dedicated to assessing various protocols for OP with a primary focus on the distal or middle rectum.

## Method

We conducted a comprehensive search of electronic databases, including PubMed/MEDLINE and Google Scholar to identify published articles and abstracts reporting on randomized clinical trials investigating OP approaches for patients with rectal cancer. We also reviewed data presented at the LUCARRE Congress held in Nice on November 25, 2023, highlighting ongoing and recently published randomized trials centered on OP.

Subsequently, the collected data were classified into three distinct categories:

1. Completed recent randomized trials with OP as the primary endpoint.
2. Randomized trials centered on local excision.
3. Ongoing trials with OP as the primary endpoint.

We also reviewed recent large-scale ‘Key randomized trials’ evaluating OS as the primary outcome.

## Results

Analyzing various randomized trials is challenging due to substantial differences among them in multiple aspects: inclusion criteria, baseline classification, staging methods (such as clinical examination, endoscopy, imaging, and biology), and details of treatment techniques, particularly in surgical procedures (including standard TME or local excisions [LE]). The differences extend to radiotherapy techniques and delineation volume. Moreover, discrepancies persist in outcome measures. The evaluation of toxicity and quality of life lacks standardized approaches. The ultimate challenge lies in assessing the crucial endpoint of the toxicity/benefit ratio.

Despite these limitations, the following comments could be made:

### Overview of recent key trials with survival as the main endpoint

These phase III trials (Table 1), in the context of LARC, aimed to optimize chemotherapy use to increase survival rates by minimizing distant metastasis. OP remains a secondary endpoint in these investigations. The key message from these trials involving a total of 3791 patients is to show that at 5 years the OS rate is nowadays, for patients with non-metastatic LARC, close to 80% which is a considerable improvement when compared to the 50–60% rate published in the 1990s [8, 9]. The observed survival benefit is likely attributable to better detection of metastases at baseline, advancements in TME surgery, reduced postoperative mortality and improved management of metastases.

**Table 1:** Key trials.

		PRODIGE 23 [8]	RAPIDO [9]	PROSPECT [7]	POLISH-II [13]	STELLAR [14]
Principal investigator		T. Conroy	RR. Bahadoer	D. Schrag	K. Bujko	J. Jin
Phase		III R	III R	II/III R	III R	III R
Number of patients		461	920	1128	515	599
Inclusion criteria		Age 18–75 y, cT3–4, M0 rectal adenocarcinoma, < 15 cm from AV	Age > 18 y; cT4a or cT4b or N2 rectal adenocarcinoma, with extramural invasion or mesorectal invasion, M0	Age > 18 y; cT2N1, cT3N0–1, mid and upper rectal adenocarcinoma, no distal rectal	Age 18–75 y, cT3–cT4 unresectable rectal cancer or unresectable local recurrence M0 ≤ 15 cm from AV	Age 18–75 y, cT3–T4 and/or N+ M0 rectal adenocarcinoma, the distal or middle third of the rectum
Design	Experimental arm	FOLFIRINOX (×6) then CAP50 then TME surgery and adjuvant chemotherapy: FOLFOX or CAPOx	SCRT (5 × 5 Gy) then CAPOx or FOLFOX chemotherapy then TME surgery	FOLFOX (×6) evaluation: Tumor size reduction ≥20% TME ± FOLFOX (×6) Tumor size reduction <20% CRT then TME ± FOLFOX (×6)	SCRT(5 × 5 Gy) then FOLFOLX ×3 then TME surgery (Group A)	SCRT (5 × 5 Gy) then CAPOX then TME surgery then adjuvant CAPOx
	Control arm	CAP50 then TME surgery then adjuvant chemotherapy: FOLFOX or CAPOx	CAP50 then TME surgery ± adjuvant chemotherapy: FOLFOX or CAPOx	CRT then TME surgery + /- FOLFOX (×8)	CRT 50.4 Gy then TME surgery (Group B)	CRT 50 Gy then TME surgery then adjuvant CAPOx ×6
Primary endpoint		DFS (3 y)	DrTF (3 y)	DFS	Pelvic R0 resection	DFS (3 y)
Results		76% experimental arm versus 69% control arm HR 0.69 p:0.034	23.7% experimental arm versus 30.4% control arm HR 0.75 p: 0.01	80.8% experimental arm versus 78.6% control arm HR: 0.92 p:0.004	77% experimental arm versus 71% control arm p:0.07 OR 1.42	64.5% experimental arm versus 62.3% control arm HR: 0.88 p<0.001
Overall survival		81.9% experimental arm versus 76.1% control arm (7 y) HR: 0.73, p: 0.03	82% experimental arm versus 80% control arm (5 y) HR: 0.9, p:0.5	89.5% experimental arm versus 90.2% control arm (5 y) HR: 1.04	73% experimental arm versus 65% control arm (3 y) HR 0.73 p 0.04	86.5% experimental arm versus 75.1% control arm (3 y) p: 0.03
Organ preservation		<5%	<5%	<5%	<5%	<5%
Pathological complete response		28% experimental arm versus 12% control arm	28% experimental arm versus 14% control arm	21.9% experimental arm versus 24.3% control arm	12% experimental arm versus 16% control arm	21.8% experimental arm versus 12.3% control arm
Disease free survival		67.6% experimental arm versus 62.5% control arm (7 y) p 0.048	23.7% experimental arm versus 30.4% control arm (DrTF) HR 0.75, p: 0.01	80.8% experimental arm versus 78.6% control arm HR: 0.92 p:0.004	53% experimental arm versus 52% in control arm HR 0.96 p 0.82	64.5% experimental arm versus 62.3% control arm HR: 0.88, p<0.001

R: Randomized; y: year; m: months; AV: anal verge; CAP50: concurrent capecitabine and 50 Gy radiation therapy; TME: total mesorectal excision; CAPOx: Capecitabine and oxaliplatin; DFS: Disease-free survival; HR: Hazard Ratio; SCRT: short course radiation therapy; DrTF: Disease-related treatment failure; OR: Odds ratio.

The findings from the PRODIGE 23 trial suggest that this benefit is closely associated with the use of the FOLFIRINOX regimen [8]. The RAPIDO trial, using consolidation chemotherapy with FOLFOX after a short course radiotherapy (SCRT), shows survival improvement but with an increase in local relapse in the SCRT arm [9]. Down staging is also observed after SCRT followed by delayed surgery, as demonstrated by the results of the Stockholm III trial [15]. Neoadjuvant chemoradiotherapy (CRT) appears to be more effective than SCRT in achieving tumor sterilization, as shown by the NACRE trial, where the rate of pathological complete response (ypCR) is 6.1% using SCRT versus 17.4% for CRT with concurrent capecitabine (CAP50) [16]. This difference may be attributed to the longer interval before surgery in the CRT arm. The PROSPECT trial highlights the potential of neoadjuvant chemotherapy with FOLFOX alone to achieve 21.9% ypCR with the possibility of avoiding radiotherapy before surgery [7]. These promising results could be due to the selection of tumors in the upper and middle rectum with the exclusion of distal rectal cancers which are more aggressive [17]. This study suggests that upper rectal cancers might be treated similarly to sigmoid cancer, using only surgery, this approach could preserve more of the rectum, maintain good bowel function, and avoid the toxicities associated with chemotherapy.

### Overview of past Phase III trials for organ preservation

A review of previously published randomized trials (Table 2) highlights several findings particularly when focusing on the endpoint of OP: Three trials explored the contribution of total neoadjuvant treatment (TNT), including the OPRA, GRECCAR12, and CAO/ARO trials. In the OPRA trial initiating treatment with CRT followed by consolidation chemotherapy has demonstrated superior outcomes in terms of complete clinical response (cCR), ypCR, and OP rates compared to induction chemotherapy [10]. The 5-year TME-free survival rate was 54% when TNT started with CRT versus 39% with induction chemotherapy. Similar findings in favor of consolidation chemotherapy were observed in the CAO/ARO/AIO-12 trial, with an increased ypCR of 28% with CRT first compared to 21% with TNT initiated with induction chemotherapy [19]. In both trials, the delay between radiotherapy and surgery was longer when starting with radiotherapy, which may provide a higher rate of cCR at the time of clinical or pathological tumor response assessment. In the GRECCAR 12 trial (NCT02514278), the use of induction chemotherapy with FOLFIRINOX before CRT (CAP50) has shown promise in improving OP rates in cT1–3 tumors measuring 4 cm or less with 71% of OP in the FOLFIRINOX group (V. Vendrely; ESTRO congress, May 2024). This encouraging rate of OP was achieved using LE after TNT with acceptable toxicity and good bowel function. However, this approach is associated with increased early toxicity due to FOLFIRINOX and is therefore limited to patients aged 75 and younger.

**Table 2:** Past trials.

		OPRA 13–213 [10]	WW2 [20]	GRECCAR 12 (V. Vendrely; ESTRO congress, May 2024)	OPERA [5]	CAO/ARO/AIO-12 [19]	KIR TRIAL [18]	NACRE [16]
Principal investigator		J. Garcia Aguilar	ALAppelt	E Rullier	JP Gérard	E. Fokas	A. Garant	E François
Phase		II R	II	III	III	II R	II	IIIR
Number of patients		324	107	218	141	306	180	102
Inclusion criteria		Age > 18 y; Middle/Distal, stage II-III(cT3–T4 N0) rectal adenocarcinoma	Age > 18 y cT1–3b N0–N1 ≤ 4 cm; Unfit for TME (PS: 0–2)	Age > 18 y; cT2–T3, cN0–1, M0 (Tumor size < 4 cm, < 10 cm to AV) mid and distal rectal adenocarinoma	Age >18 y; cT2, cT3a-b, < 5 cm, mid and distal rectal adenocarcinoma	CT3–4 and/or N+; distal or mid rectal adenocarcinoma	Operable cT2 or T3 and: node-positive (N+), close MRF, or extramural venous invasion (EMVI+).	≥75 years Resectable cT3–T4 rectal or cT2 of the very low rectum
Design	Experimental arm	Induction CT then CRT (CAP54 or 5FU) restaging: cCR or nCR: WW Incomplete response: surgery	CRT 62 Gy (tumor boost) + 50.4 Gy (Lymph node)	Induction CT FOLFIRINOX (×4) + CRT (CAP50) ± local excision	CRT (CAP45)+CXB 90 Gy (Group B)	Induction CT then CRT Then TME (Group A)	Neoadjuvant CT then HDRB then surgery + adjuvant FOLFOX ×6 (Arm A)	SCRT 5 × 5 Gy + TME
	Control arm	CRT (CAP54 or 5 FU) then consolidation CT FOLFOX or CAPOX restaging: cCR or nCR: WW Incomplete response: surgery	-	CRT(CAP50) ± surgery	CRT (CAP45)+ EBRT Boost 9 Gy (group A)	CRT then consolidation CT 3FOLFOX then TME (Group B)	HDRBT then surgery then adjuvant FOLFOX ×12 (Arm B)	CRT 50 Gy + TME
Primary endpoint		DFS at 3 years with OP	OP without surgery at 2 years	OP rate and absence of stoma at 1 year	OP without progressive local disease at 3 y	pCR	CT compliance	Pelvic R0 resection rate
Results primary endpoint		76% experimental arm versus 75% historical group	61%	71.7% experimental arm versus 62.7% control arm p 0.056	81% experimental arm versus 59% control arm HR 0.36 p:0.002	25% group B versus 17% group A (Only group B fulfilled the statistical hypothesis)	80% Arm A versus 53% Arm B p:0.002	84.3% experimental arm versus 88% control arm p: 0.28
Organ preservation		53% (Control arm) versus 41% (Experimental arm) p: 0.01	61%	71.7% (Experimental arm) versus 62.7% (control arm) p 0.056	81% group B (97% T<3 cm) versus 59% group A (control arm)	0%	0%	<5%
Clinical complete response		58% (Control arm) versus 51% (Experimental arm)	86%	-	92% in group B versus 63% in group A	-	-	-
Pathological complete response		-	-	48% in experimental arm versus 28% in control arm	-	25% group B versus 17% group A	30.5% Arm A versus 28.3% Arm B	17.4% control arm versus 6.1% experimental arm
Survival (OS, DFS)		DFS (5 y) 71 %experimental arm versus 69% control arm p: 0.6 OS (5 y) 88% experimental arm versus versus 85 %control arm)	-	OS 95% 3 y DFS 86% 3 y (no significant difference between arms)	DFS 83% 3 y (no significant difference between arms)	73% in both arms	DFS 72.3 Arm A versus 68.3% Arm B 5 y OS 83.8% Arm A versus 82.2% Arm B	OS 85% experimental arm versus 80%control arm (3 y) p: 0.06 DFS 80% versus 85% (3 y)

R: Randomized; y: year; m: months; CRT: concurrent chemoradiation therapy; 5FU: 5 fluorouracil; CAP: Capecitabine; CAP54: capecitabine with concurrent radiation therapy 54 Gy; CAPOX: capecitabine and oxaliplatin; WW: watch and wait; cCR: complete clinical response; nCR: near complete clinical response; SCRT: short course radiation therapy; TME: total mesorectal exicison; OP: organ preservation; ypCR: pathological complete response; DrTF: Disease related treatment failure; HR: Hazard Ratio; DFS: Disease free survival; EBRT: external beam radiation therapy; HDRBT: High dose rate brachytherapy; MRF: mesorectal fascia.

Two studies investigated radiotherapy dose escalation: The Danish WW2 trial showed the feasibility of escalating EBRT (external beam radiation therapy) doses with a boost to 62 Gy in early-stage tumors, achieving an OP rate of 61% with an acceptable toxicity level [20]. Building upon these promising findings, the ongoing WW3 Phase III trials aim to validate the efficacy and safety of dose-escalated EBRT in a larger patient cohort. In the OPERA trial, the use of contact X-ray brachytherapy (CXB) with chemo-radiotherapy (CAP45) has emerged as a potent strategy to enhance treatment outcomes in rectal cancer. This sequential approach has shown high rates of cCR and OP, particularly in T2–T3a/b tumors measuring less than 3 cm, with OP rates over 90% when starting treatment with CXB [5].

One of the key takeaways from this data is the increasing acceptance of OP as a viable strategy for operable patients. A crucial first step, if OP is planned, is to achieve a cCR.

### Overview of local excision trials

Clinical trials on LE with planned operative procedures (Table 3) are specifically targeting early rectal cancers classified as cT1–T2 or T3a/b < 4 cm, N0. In the ACOSOG Z6041 trial, 4% of patients experienced local recurrence, and 88% were alive and free from disease at 3 years with 91% OP [21]. The authors concluded that LE following CRT could serve as an alternative to TME surgery for unfit patients. The findings from the GRECCAR 2 trial, which reported a 5% local recurrence rate in the local excision group, further support these conclusions and demonstrated that at 5 years LE after CRT did not negatively impact OS compared to TME [22].

**Table 3:** Local excision trials.

		CARTS[21]	ACOSOG Z6041[18]	TREC[20]	GRECCAR 2[19]	NEO TRIAL[22]	TAU-TEM TRIAL[6]
Principal investigator		RCH. Stijns	J. Garcia Aguilar	S. Bach	E. Rullier	Hf. Kennecke	X. Serra-Aracil
Phase		II	II	R. Feasibility study	III R	II	III R non-inferiority
Number of patients		55	79	55	148	58	162
Inclusion criteria		Age > 18 y; rectal adenocarcinoma up to 10 cm from AV; cT1–3; N0	Age > 18 y; T2N0 distal rectal adenocarinoma; tumor size< 4 cm	Age > 18 y;T1–2 N0 rectal adenocarinoma; tumor size <3 cm	Age > 18 y;T2–3 rectal adenocari-noma; size < 4 cm; < 8 cm to AV; residual tumor after CRT ≤ 2 cm	Age > 18 ycT1–T3ab N0 mid to low-rectal adenocarcinoma eligible for endoscopic resection	Age > 18 y, rectal adenocarcinoma up to 10 cm from AV, superficial T2–T3a/b–N0; lesion ≤4 cm diameter
Design	Experimental arm	CAP50 then TEM if good response or TME if no response	Neoadjuvant CRT then local excision	SCRT 5 × 5 Gy then TEM	Local excision	Neoadjuvant CT then local excision	CAP50 then local excision
	Control arm			TME	TME		TME
Primary endpoint		Number of patients with minimal residual disease	DFS (3 y)	Cumulative recruitment of randomized patients (12, 24, and 36 months)	5-year oncological outcomes of local recurrence, metastatic disease, DFS, OS	OP rate at 3 years	Local recurrence
Pathological complete response		38%	44%	30%	40%	56%	44.3%
Results		OS:81.6% DFS: 82.8% OP: 50–70%	DFS: 88.2% OP: 91%	OS: 93% (no significant difference) DFS: 85% (no significant difference) OP 70%	Local recurrence: 7% both arms DFS: 70% versus 72% p:0.6 OS: 84% versus 82% p:0.8 OP: 45%	ypT0: 13% ypT0 or pT1 57% when including pT2 who declined TME OP 79%	pCR 44.3% experimental arm 82.7% OP experimental arm at 1 year OP 82.7%

y: years; AV: anal verge; CAP50: chemotherapy capecitabine and concurrent radiotherapy 50 Gy; TEM: trans-anal endoscopic microsurgery; TME: total mesorectal excision; OS: overall survival; DFS: Disease-free survival; OP: organ preservation; CRT: chemoradiation therapy; R: Randomized; SCRT: short course radiation therapy; CT: chemotherapy.

The TREC and CARTS trials achieved similar OP rates: 50–70% in the CARTS trial and 70% in the TREC trial, using LE after irradiation [23, 24]. The rate of ypCR was higher with a CRT than with a SCRT (Table 3).

A similar OP rate (33/58: 79%) was reported in the Canadian NEO trial using neoadjuvant chemotherapy alone with lower ypCR (8/58: 17%) [25]. The Spanish TAU-TEM study was the only phase III trial to compare, for (cT1) cT2–T3 a/b N0, CRT (EBRT: 50.4 Gy with capecitabine) followed by LE versus TME alone. In the LE group, ypCR was 44% (35/77), and the overall OP rate reported was 82.7%. No results were reported regarding the 3-year outcomes, local recurrence rates, or bowel function assessments. In the TME group, the pN1 rate was 21% (17/81), indicating that for cT1N0 tumors, including T1sm3, the pN1 rate remains below 20%. However, for T2N0 tumors, mesorectal irradiation, preferably with CRT, is essential to mitigate the high risk of perirectal nodal relapse following local excision or intra-rectal irradiation [6].

### Overview of the ongoing trials aiming at organ preservation

All these trials (Table 4) were presented and discussed during the November 2023 LUCARRE meeting in Nice. They specifically focus on four critical aspects regarding strategies for OP in the management of rectal cancer:

**Table 4:** Ongoing trials.

		CCHOWW	JANUS	OPAXX	STAR-TREC	MORPHEUS	APHRODITE	TRESOR	WW3	ACO/ARO/AIO-18.1
Principal investigator		RO. Perez	J. Joshua Smith	B. Grotenhuis, P. Burger	S. Bach	T. Vuong	S. Noutch	J. Durand-labrunie	LH. Jensen	C. Roedel
Phase		III R	II/III R	II	IIR	III	IIR	III	IIR	III
Number of Patients		216	312	168	120	145	104	212	112	702
Inclusion Criteria		T2–T3 N0–1 rectal adenocarcinoma; lower edge of tumor at the level or below the anorectal ring	cT4N0 or any cTN+ rectal adenocarcinoma distant < 12 cm from the anal verge	Rectal adenocarcinoma within 10 cm to AV; nCR or a small residual tumor mass less than 3 cm after CRT	cT1–3bN0 (≤5 mm mesorectal invasion) rectal adenocarinoma or uT1–uT3b; tumor size < 4 cm	cT2–T3, N0–N1 (<5 mm mesorectal invasion)	cT1–T3b, ≤ 5 cm, N0, non eligible to TME	cT2N1 or cT3N0–1, M0 rectal adenocarinoma; <11 cm to AV; Size ≥ 3.5 cm and ≤ 6 cm	cT1–T3, rectal adenocarcinoma size≤ 4,5 cm; candidate to rectal resection	cT3–4, N+ rectal adenocarinoma 0–12 cm to AV, CRM+ (<1 mm), EMVI+
Design	Experimental arm	CAP54 followed by consolidation CT with 5 fluorouracil and oxaliplatin then evaluation if cCR: WW	CAP54 followed by consolidation triplet (FOLFIRINOX) chemotherapy then evaluation	CXB boost	OP with SCRT 5 × 5 Gy then response evaluation (ARM A)	CAP45 + EBRT boost 9 Gy (arm A)then response evaluation	CRT 62 Gy on tumor, 50,4 Gy on the mesorectum	Induction chemotherapy FOLFIRINOX + CXB + CAP50 then evaluation: surgery or WW	CRT 62 Gy on Tumor and 50.4 Gy on elective volume	CAP 54 Gy then consolidation CT the evaluation Surgery or W&W
	Experimental arm				OP with long course CRT then response evaluation	CAP45 + IGAEBT 30 Gy x 3then response evaluation				
	Control arm	CAP54 then 5flurouracil alone chemotherapy then evaluation if cCR: WW	CRT followed by consolidation doublet (FOLFOX or CAPOX) chemotherapy	Extending the waiting interval and local excision	TME		CRT 50,4 Gy	Induction chemotherapy FOLFIRINOX + CAP 50 then evaluation: surgery or WW	CRT 50.4 Gy	SCRT then consolidation CT then response evaluation Surgery or W&W
Primary Endpoint		The decision to WW due at 18 weeks from radiotherapy	cCR rate up to 5 y	OP at 1 y	OP at 12 months	OP at 2 years	Rate of cCR at 6 months after start of treatment	Survival with OP	OP at 2 years	OP at 3 year
Start Of Inclusion		October 2022	November 2022	April 2021	October 2017	April 2017	February 2020	2024	January 2020	January 2020
End Of Inclusion		2026	September 2024	March 2025	October 2021	January 2024	October 2024	2028	December 2027	September 2023

R: Randomized; CAP54: chemotherapy with capecitabine and concurrent radiation therapy 54 Gy; cCR: clinical complete response; WW: watch and wait; nCR: near complete response; CRT: concurrent chemotharapy and radiation therapy; CXB: contact x-ray brachytherapy; OP: organ preservation; TME: total mesorectal excision; EBRT: external beam radiation therapy; IGAEBT: adaptative brachytherapy boost; ypCR: pathological complete response; CEM: circumferentiel resection margin; EMVI: extramural vascular invasion; CRM: circumferential resection margin; SCRT: short course radiotherapy.

1. What is the optimal TNT combination for cT2/T3 N0–1 tumors?

The Brazilian CCHOWW compares, after initial CRT (CAP 54) consolidation chemotherapy with capecitabine in the control arm versus FOLFOX or XELOX in the experimental arm. The JANUS trial compares doublet chemotherapy: FOLFOX or CAPOX in the control arm versus triplet chemotherapy FOLFIRINOX in the experimental arm. The German ACO/ARO/AIO 18.1, which has already closed to accrual with 702 patients, compares TNT with CRT in the experimental arm versus SCRT in the control arm, both followed by consolidation chemotherapy with either FOLFOX or CAPOX.

2. Is it possible to increase OP by using EBRT dose escalation for T1–3 N0 tumors smaller than 4.5 cm?

The Danish WW3 and British APHRODITE trials compare a dose of 50.4 Gy in the control arm versus external radiotherapy dose escalation to 62 Gy using a simultaneous integrated boost in the experimental arm.

3. What is the optimal strategy for using local excision to achieve OP?

The STAR-TREC trial employed a patient preference method to compare three treatment arms in T1–T3b N0 tumors smaller than 4 cm: TME alone (control arm) versus SCRT or CRT (CAP50) both followed by local excision (experimental arm). The Dutch trial OPAXX selects patients after preoperative irradiation who are in near clinical response (nCR) or partial response with residual tumor smaller than 3 cm after CRT; the trial compares immediate CXB boost versus extending the waiting period followed by a local excision.

4. What is the possible role of endorectal boost using brachytherapy?

The Canadian MORPHEUS trial, a replication of the OPERA trial, uses an Iridium HDR boost instead of the CXB boost. The French TRESOR trial explores a TNT in intermediate cT2N1 or T3N0–1 tumors with a diameter exceeding 3.1 cm but less than 6 cm. After FOLFIRINOX induction chemotherapy, the trial compares CRT (CAP 50) with or without a CXB boost.

## Discussion

Some general comments can be made when analyzing these different trials: For LARC, with present-day treatments, usually combining TME radiotherapy and chemotherapy, the 5-year OS is close to 80%. One of the major challenges is actually to improve the quality of life using OP with the selective omission of TME surgery in patients who achieved cCR after multimodal treatment. The ‘Watch and Wait’ approach pioneered by Habr Gama is increasingly accepted among leading institutions worldwide for managing selected rectal cancer patients after neoadjuvant therapy. The past phase III trials aiming at OP tend to show that rectal preservation does not negatively affect long-term survival (Greccar 2 – OPRA) [5, 22]. Depending on the stage of the tumor and the treatment used, the rate of 3-year or 5-year OP is close to 50% with TNT for LARC and up to 80% for early cT2–T3 using local excision with preoperative radiotherapy and/or chemotherapy.

For tumors <3 cm, a CXB boost given as the first treatment achieves over 90% of OP [5]. In most cases, achieving a high rate of cCR – a key factor for OP – relies on radiotherapy, likely involving a high tolerable radiation dose. Recently planned OP in early tumors (cT2N0 < 4 cm) has been considered a standard strategy in French and Welsh guidelines for operable patients [26]. So far, the differences in phase III trials make it difficult to determine the best approach for OP or draw definitive conclusions regarding the optimal strategy.

The role of local excision in rectal cancer treatment needs to be clarified: Local excision may not be beneficial in cases of complete pathological response (ypT0N0), as it often lacks significant clinical advantages. It may be insufficient when removing an ypT2 or R1 tumor but it can be useful with long-term local control and OP when removing small residual lesions or minor ‘regrowth’ after neoadjuvant treatment.

The ongoing trials will certainly bring new data to optimize the OP strategy. Individualization of treatment should be possible according to the tumor at presentation (site, stage, type) and patient profile (age, frailty, stoma averse). It is likely that for early (cT1) cT2 N0, de-escalation could be possible.

A trial comparing local excision with a CXB boost in combination with a SCRT or a CRT could be designed for early lesions. The place and best regimen of chemotherapy should be better understood after the results of the ongoing trials. Chemotherapy with FOLFIRINOX, despite its known toxicity, may lead to an increased OP rate and improved OS. For LARC, it may be possible to achieve close to 80% planned OP, similar to outcomes seen in anal carcinoma. A combination of all modalities: long course CRT, consolidation or induction chemotherapy, endo-rectal radiation boost, and local excision could achieve this goal in the ‘bad’ LARC (cT3 c/d N1 with a diameter < 6 cm). However, this approach may not be effective for ‘ugly’ LARC (cT3c/d N2 with a diameter > 6 cm) where TME could remain the standard treatment unless an unexpected cCR is observed after TNT. Furthermore, at present time, from an epidemiological point of view, and with OP in mind, three points can be stressed:

1. Around 20% of rectal cancers at initial diagnosis present with detectable metastasis and chemotherapy plays a major role with most of the time an attempt at some OP.
2. With increased screening resulting in earlier diagnosis, more cT1–T2 N0 cases are detected which are the best candidates for OP.
3. With a general increase in cancer cure, around 20% of subsequent cancers occur in patients already treated for previous pelvic malignancies using radiotherapy (often for prostate cancer in men or uterine or cervical in women). In most cases, second EBRT for rectal cancer is no longer possible but localised endorectal irradiation is still available. Overall, recent data from cancer registries in Western countries show 5-year OS for all rectal cancers (M0–1) between 60 and 70%, possibly becoming better than for colon cancers [27].

Several questions remain open to debate: The role of ERUS versus MRI in distinguishing between cT1–cT2 and T3 tumors as well as determining the optimal approach for assessing tumor response and the most appropriate MRI criteria. Additionally, the role of biopsy in evaluating response, along with the potential of biomarkers or immunoscores to predict outcomes, remains a subject of ongoing investigation [28].

A systematic analysis of mismatch repair (MMR) genes is essential to recommend standard immunotherapy in cases of MMR deficiency, which is relatively uncommon in the lower rectum after the age of 50 [29]. The phase II study conducted by Andrea Cercek et al. enrolled 36 patients with MMR-deficient stage II or III rectal adenocarcinoma, who received single-agent DOSTARLIMAB, a PD-1 inhibitor, every 3 weeks for 6 months. Patients who achieved a CCR after DOSTARLIMAB therapy did not proceed to CRT or surgery. All patients (100%) demonstrated a cCR, with no signs of tumor on imaging, endoscopy, or biopsy, and no cases of progression or recurrence reported over a follow-up period of 6 to 25 months. No severe adverse events (grade 3 or higher) were observed. These findings suggest that locally advanced, MMR-deficient rectal cancer is highly responsive to PD-1 blockade, extended follow-up is necessary to confirm the long-term durability of this response [27]. For MMR proficient tumors, the suspected immuno-triggering effect of radiation therapy could be combined with immune checkpoint blockade, especially using CXB, which may not inhibit the immune process in the tumor-draining lymph nodes.

## Conclusion

In conclusion, as W. Heald recently wrote ‘It is urgent to realize that a cCR usually means that the patient may not need surgery’ and as G. Beets confirmed [30] ‘In the last two decades OP has evolved from controversy to common clinical practice’, OP is becoming a major field of clinical research for rectal cancers. Only large phase III trials will be able to bring strong evidence. It is hoped that following the past and ongoing trials presented in this overview, international cooperation will make it possible to initiate such large trials to rapidly define optimal strategies for OP and answer the priority questions for the benefit of the patients.

## Data Availability

The data that support the findings of this study are not publicly available due to restrictions related to patient confidentiality and privacy. Access to the data may be available upon reasonable request and with approval from the appropriate ethics committee.
